# Integrin α5β1 Function Is Regulated by XGIPC/kermit2 Mediated Endocytosis during *Xenopus laevis* Gastrulation

**DOI:** 10.1371/journal.pone.0010665

**Published:** 2010-05-17

**Authors:** Erin Spicer, Catherine Suckert, Hyder Al-Attar, Mungo Marsden

**Affiliations:** Department of Biology, University of Waterloo, Waterloo, Ontario, Canada; University of Western Ontario, Canada

## Abstract

During *Xenopus* gastrulation α5β1 integrin function is modulated in a temporally and spatially restricted manner, however, the regulatory mechanisms behind this regulation remain uncharacterized. Here we report that XGIPC/kermit2 binds to the cytoplasmic domain of the α5 subunit and regulates the activity of α5β1 integrin. The interaction of kermit2 with α5β1 is essential for fibronectin (FN) matrix assembly during the early stages of gastrulation. We further demonstrate that kermit2 regulates α5β1 integrin endocytosis downstream of activin signaling. Inhibition of kermit2 function impairs cell migration but not adhesion to FN substrates indicating that integrin recycling is essential for mesoderm cell migration. Furthermore, we find that the α5β1 integrin is colocalized with kermit2 and Rab 21 in embryonic and XTC cells. These data support a model where region specific mesoderm induction acts through kermit2 to regulate the temporally and spatially restricted changes in adhesive properties of the α5β1 integrin through receptor endocytosis.

## Introduction

Cell adhesion is central to many biological processes including development, cancer metastasis, and wound healing. Integrin heterodimers are key regulators of cell adhesion and the interaction of cell surface integrin receptors with the extracellular matrix (ECM) has been well characterized [1]. Most integrins exist on the cell surface in a low affinity state and through various stimuli can be activated to a high affinity state that promotes cell adhesion [2]. Once activated, integrins are capable of promoting both ECM assembly as well as cell migration on ECM substrates. However, the presence of activated integrins at the cell surface is not sufficient to drive cell migration and there is growing evidence that endocytic recycling of activated integrins is a key step in regulating cell adhesion [3].

In the *Xenopus laevis* embryo the α5β1 integrin plays a number of critical roles during gastrulation. Cells of the blastocoel roof use α5β1 to assemble a fibronectin (FN) matrix just prior to gastrulation [4]–[6]. Upon the initiation of gastrulation involuted mesoderm cells use α5β1 integrin to adhere and migrate directionally on this FN matrix [7]–[9]. As the expression of α5β1 integrin is ubiquitous in the *Xenopus* embryo, the differential use of α5β1 by ectoderm, endoderm and mesoderm suggests that this integrin exists in multiple activation states. Animal cap ectodermal cells adhere to the Arg-Gly-Asp (RGD) sequence of Central Cell Binding Domain (CCBD) of FN. Treatment of ectodermal cells with activin induces a mesodermal cell fate and results in cell spreading and migration on FN [10] using the RGD sequence in conjunction with the neighboring synergy site [5], [11], [12]. The spreading and migration of activin-treated ectodermal cells on FN occurs with same temporal regulation as observed in involuted mesoderm cells, indicating that in the embryo activation of the α5β1 integrin is under strict temporal and spatial regulation [6]. Several lines of evidence point to the cytoplasmic domains of both the α and β integrin subunits as being required for integrin activation in *Xenopus*. Expression of a β1 cytoplasmic domain dominant negative construct interferes with both FN assembly and activin induced cell migration [13]. Chimeric integrin molecules consisting of the α4 extra-cellular domain and a variety of α subunit cytoplasmic domains reveal that while a number of α subunit cytoplasmic domains can substitute in cell adhesion, the α6 and α5 cytoplasmic domains are uniquely necessary for FN assembly [14].

As α5 and α6 cytoplasmic domains confer the ability to assemble FN and both contain a C terminal Class I PDZ binding motif we decided to ask what role this domain plays in *Xenopus* development. GIPC, a PDZ domain-containing protein, has previously been identified as interacting with the PDZ binding motif of the mammalian α5 and α6 integrin subunits [15], [16]. GIPC has been demonstrated to interact with multiple trans-membrane proteins with Class I PDZ binding motifs including Tax [17], TrkA [18], Glut-1 [19], SemaF [20], neuropilin [21], syndecan [22], gp75 [23], and the NMDA receptor [24]. GIPC also acts as a scaffolding protein, interacting with itself and other proteins through regions outside its PDZ domain. Recently, Valdembri et al. (2009) demonstrated that GIPC provides a link between the VEGF co-receptor neuropilin and α5β1 integrin in endothelial cells. The interaction of nrp-1, α5β1, and GIPC results in the rapid turnover of activated α5β1 in migrating cells [21]. Their results suggest that GIPC may function to cluster cell surface receptors within specific domains effectively compartmentalizing the components of signal transduction pathways.

In *Xenopus* the only studies examining the role of GIPC addressed the interaction between XGIPC/kermit2 and the IGF receptor [25], [26]. Here we look at the interactions between kermit2 and the α5 and α6 integrin subunits. We demonstrate that kermit2 binds the cytoplasmic domain of the α5 and α6 integrin subunits, and that the interaction with α5β1 results in receptor endocytosis during activin induced cell migration.

## Results

The adhesive activity of the α5β1 integrin is regulated in both time and space during *Xenopus* development. This change in integrin activity can be attributed in part to the cytoplasmic domain of the α subunit. To understand the molecular mechanisms behind this regulation we asked what molecules known to interact with the cytoplasmic domain of α5 are also expressed during gastrulation. One of the molecules fitting these criteria was XGIPC/kermit2, which has previously been implicated in the regulation of IGF signaling [25].

### XGIPC/kermit2 Interacts with The α5 and α6 Integrin Subunits

It has previously been reported that GIPC binds to the C-terminal region of the cytoplasmic domain of the mammalian α5 and α6 integrin subunit [15], [16]. To characterize the potential interactions between kermit2 and *Xenopus* α integrin subunits we performed yeast two hybrid assays. We generated pEG-202 derived bait plasmids expressing either full-length kermit2, or a version of kermit2 in which the central PDZ domain ALGL sequence had been altered to AEEL (Kermit2_mut_). Bait plasmids were expressed in combination with pJG4-6 derived prey plasmids expressing the cytoplasmic domains of *Xenopus* integrin α5, α6, or αV. Putative PDZ interacting consensus sequences are found in both the integrin α5 (ASEA) and α6 (TSDA) subunits (Table 1 bold). We used the αV cytoplasmic domain as a negative control as it does not contain a putative PDZ interacting domain (Table 1).

**Table 1:** Amino acid sequences of *Xenopus* α integrin cytoplasmic domains.

α5 KVGFFKRSYQYGTAMEKAELKPQA**ASEA**
α6 KVGFFRRDKKDAQFDATYHKAEIHAQPSDKERL**TSDA**
αV KVGFFKRVRPPQEETEREQLQPQENGEGITDT

**Figure 1:**
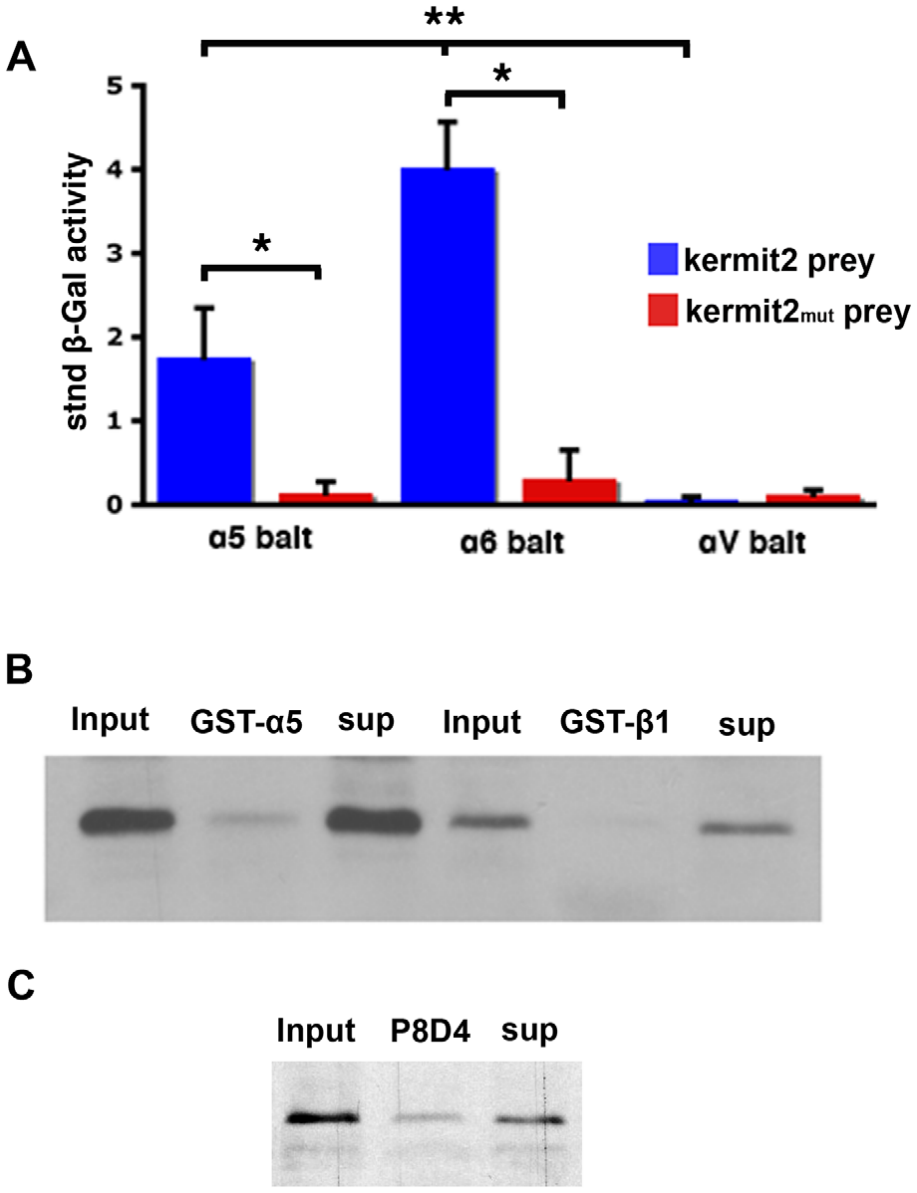
Kermit2 interacts with the cytoplasmic domain of the α5 and α6 integrin subunits. (A) Yeast two two-hybrid assays were conducted using α5, α6 and αV cytoplasmic domains as bait in combination with kermit2 or kermit2_mut_ as prey. The data is presented as average normalized β-galactosidase activity (±SD). The interaction of kermit2 with α5 and α6 is abolished by the AEEL mutation in kermit2_mut_ (* P<0.002). The αV subunit does not interact with kermit2 or kermit2_mut_ (** indicates P<0.001 between α5 or α6 and αV bait constructs). (B) GST pulldowns. HA tagged Kermit2 is detected in lysates (lane 1 input), and is pulled down with a GST-α5 fusion construct (lane 2 GST-α5). Most of the kermit2 remains in the supernatant (lane 3 sup). A control GST-β1 construct (lane 5 GST-β1) does not pull Kermit2 from lysates (lane 4 input, lane 6 sup). (C) Coimmunoprecipitation of kermit2 with α5β1 integrin. HA tagged kermit2 is detected in lysates (lane 1 input). Kermit2 is found in association with immunoprecipitated α5β1 (lane 2 P8D4), while a significant portion of kermit2 remains in the supernatant (sup).

A robust interaction between kermit2 and the α5 and α6 subunits was observed in two hybrid assays. This interaction is abolished when the central PDZ domain of kermit2 is mutated from ALGL to AEEL (kermit2mut). As expected there was no observable interaction between kermit2 and the αV cytoplasmic domain (Figure 1A). The interaction between the α6 cytoplasmic domain is stronger than that with the α5 cytoplasmic domain and likely reflects the differences between the SEA and SDA motif as steady state expression levels of both bait and prey constructs are similar in all transformants (Figure S1). As the α6 subunit is not expressed during gastrulation we concentrated our efforts on further characterizing the α5 subunit interaction.

Given the interaction between kermit2 and the α5 cytoplasmic domain in the yeast two-hybrid system we next asked if such an association occurs in embryos. We used GST fusion constructs encoding *Xenopus* α5 and β1 cytoplasmic domains to probe embryo lysates obtained from stage 12 embryos expressing HA tagged kermit2. The GST-α5 construct pulled down small amounts of HA tagged kermit2. As expected the GST-β1 construct showed no interaction with Kermit2 (Figure 1B). We then asked if integrin α5 interacted with kermit2 in vivo. When we over expressed HA tagged kermit2 we could pull down kermit2 with an antibody directed against α5β1 integrins (Figure 1C). Our results suggest that kermit2 is able to interact with α5β1 in vivo.

### Kermit2 is Required for FN Matrix Assembly

**Figure 2:**
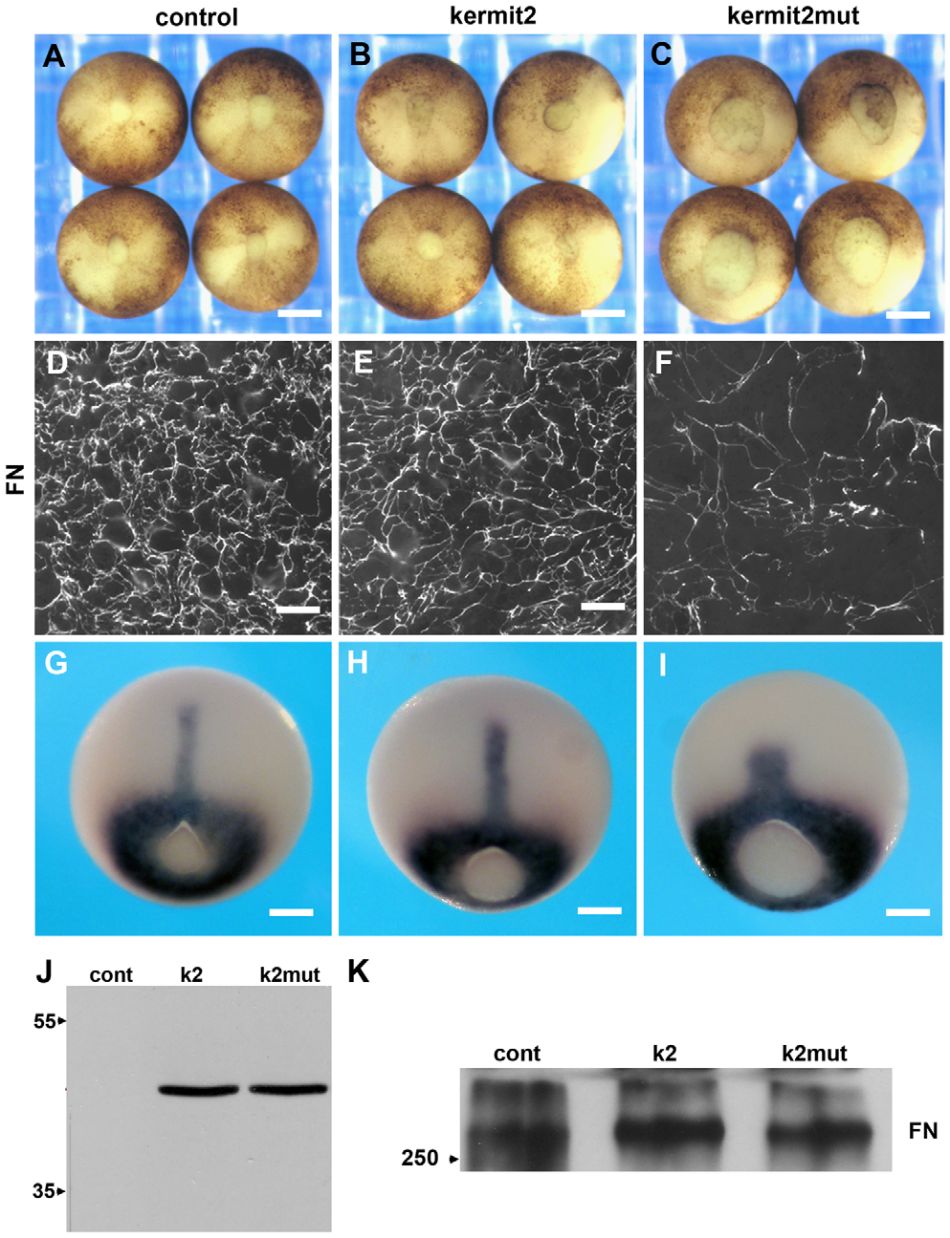
Kermit2_mut_ inhibits *Xenopus* gastrulation. (A) Control embryos injected with water close their blastopores and (D) assemble a dense FN matrix. (B) Embryos injected with kermit2 also gastrulate and (E) assemble FN matrix normally. (C) Embryos injected with kermit2mut mRNA fail to close the blastopore and (F) have a sparse FN matrix. (G–I) Xbra in situ hybridizations indicate normal mesodermal patterning in (G) control embryos, as well as (H) embryos expressing kermit2, and in embryos expressing (I) kermit2mut. Note that axial extension is inhibited in (I). (J) Western blot demonstrating equal expression of HA tagged kermit2 and kermit2 constructs. Embryos injected with water (cont) do not express the construct, while embryos injected with RNA encoding kermit2 (k2) and kermit2mut (k2mut) express equal amounts of either construct. Molecular mass markers are indicated on the right of the panel. (K) Kermit2mut expression does not inhibit FN protein accumulation. Western blots demonstrate that there is no substantial change in FN protein expression (FN) in water injected embryos (cont), embryos expressing kermit2 (k2), or embryos expressing kermit2mut (k2mut). Molecular mass markers are indicated on the right of the panel. (A–C) size marker  = 200 µM. (D–F) size marker  = 30 µM. (G–I) size marker 100 µM.

Having established that kermit2 interacts with the α5 cytoplasmic domain we then asked what role kermit2 may play in development. The gastrula stage *Xenopus* embryo provides a convenient assay for α5β1 integrin function as both the assembly of FN and the cell movements that depend upon FN matrix are well characterized [27]. We over expressed kermit2 and kermit2mut in *Xenopus* embryos and monitored blastopore closure, a process dependant upon α5β1 integrin activity and FN matrix assembly [13]. Embryos over-expressing kermit2 close their blastopores normally and assemble a dense FN ECM (Figure 2B, F). Kermit2mut acts as a dominant negative as embryos fail to close their blastopores, and have a dramatic decrease in FN matrix lining the blastocoel roof (Figure 2C, F). Taken together these results indicate that normal kermit2 function mediated through the PDZ binding domain is required for FN matrix assembly and gastrulation. In situ hybridizations with an Xbra probe reveal that defects in gastrulation are not due to deficits in mesodermal patterning but likely stem from defects in convergent extension due to a disrupted FN matrix (Figure 2G–I). Kermit2 and kermit2mut expressing embryos have similar amounts of FN indicating that the lack of FN matrix assembly is not due to a decrease in FN protein abundance (Figure 2K). RT_PCR demonstrates no change in gene expression levels for EF1α, chordin, Xbra, FN, or integrin α and β subunits (Figure S2). Therefore, we conclude that the defects in gastrulation can be attributed to a loss of integrin-mediated assembly of FN.

**Figure 3:**
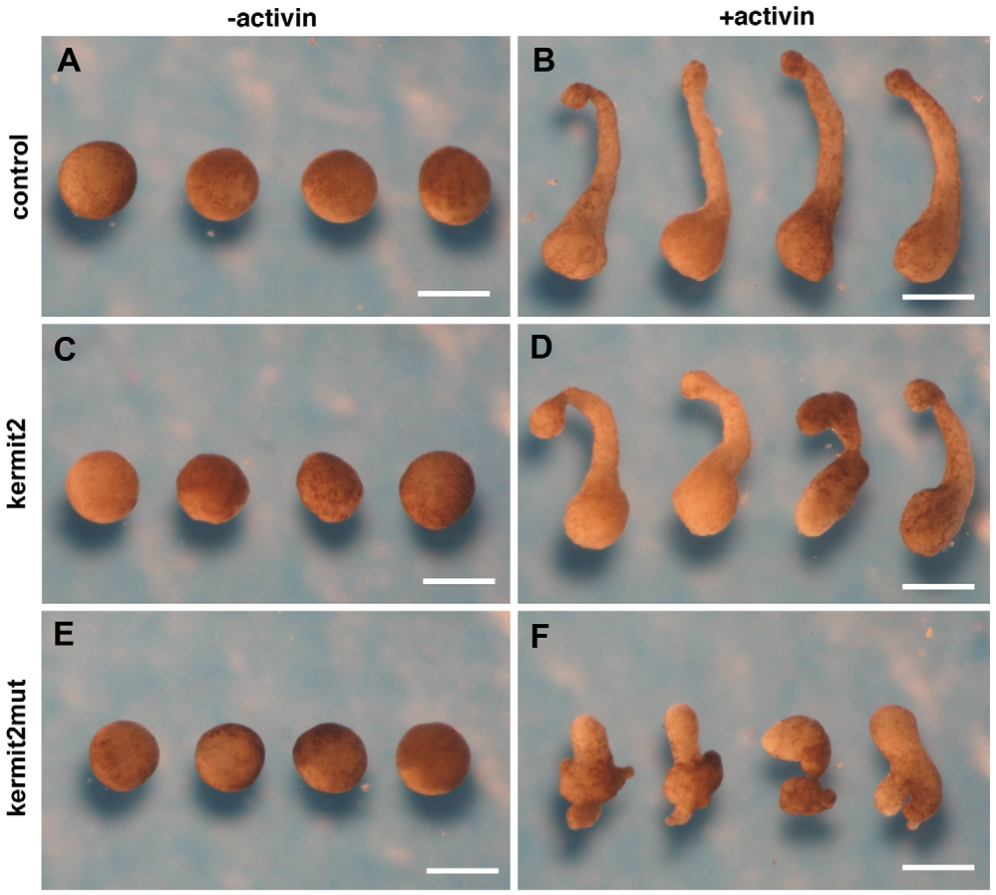
Animal cap assays (A, C, E) minus activin or (B, D, F) plus activin. (A, B) Control activin-treated animal caps extend. (C, D) Kermit2 has no effect on activin-treated animal cap extension. (E, F) Kermit2mut inhibits activin-treated animal cap elongation. Size marker  = 100 µM.

We then used animal cap assays to confirm these results. Animal caps isolated from control and kermit2 expressing embryos extend via convergent extension in the presence of 20 ng activin (Figure 3A–D). Caps isolated from embryos over expressing kermit2mut fail to extend in the presence of activin (Figure 3E–F). Thus, kermit2 is required for FN matrix assembly and the cell movements associated with convergent extension.

### Kermit2 Morpholino Knockdowns

**Figure 4:**
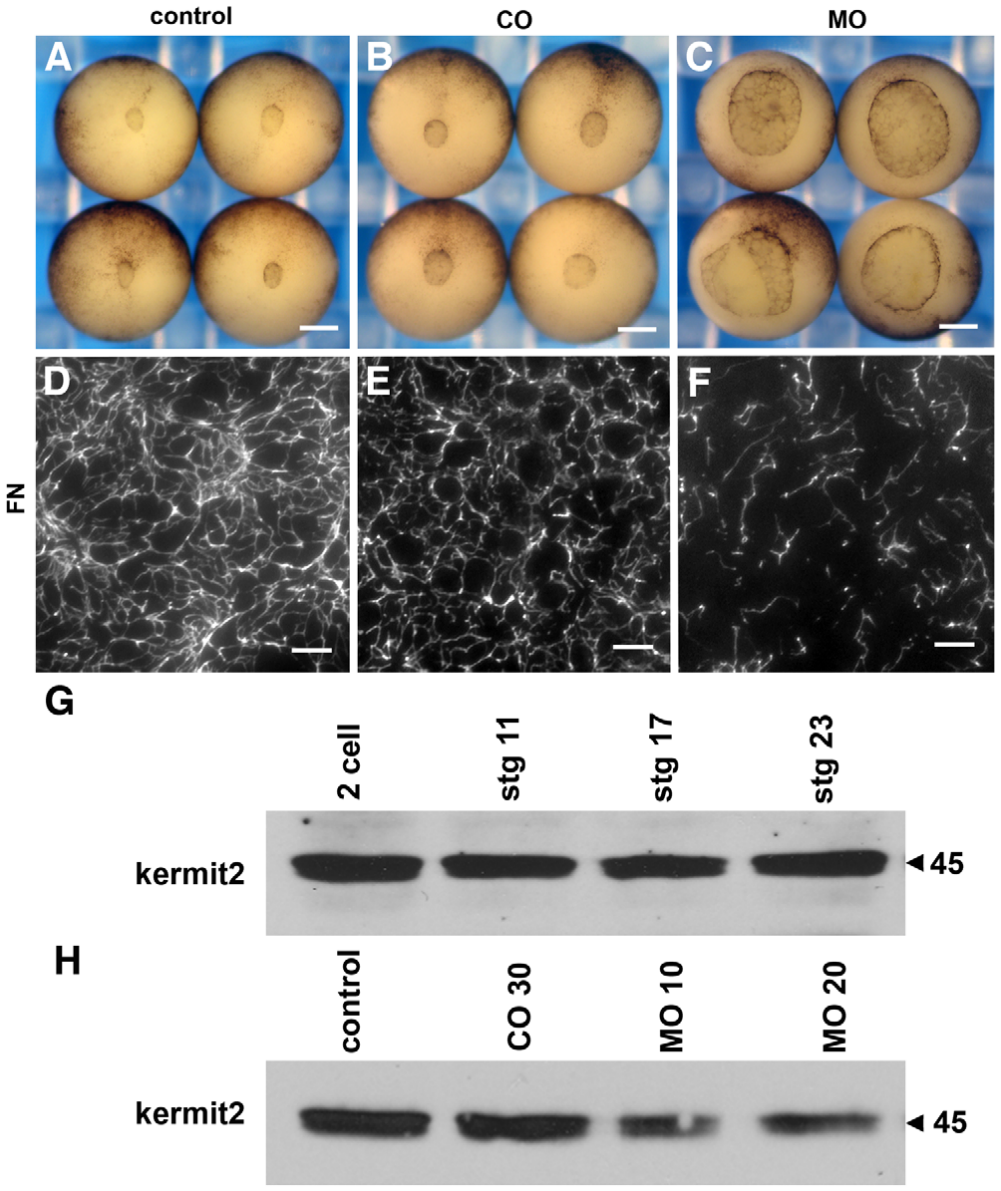
Morpholino knock down of kermit2 inhibits gastrulation and FN matrix assembly. (A–C) Ventral view of stage 12 embryos. (D–F) Late gastrula stage BCR stained for FN. (A) Stage 12 control embryos gastrulate normally and (D) assemble a dense FN matrix. Embryos injected with 20 ng of Kermit2 5 mismatch control morpholino (COMO; B) gastrulate normally, and (E) exhibit a small decrease in FN matrix assembly. (C) Embryos injected with kermit2 inhibiting morpholino (MO) exhibit delayed blastopore closure and (F) a sparse FN matrix network. (G) Western blot of kermit2 expression. Kermit2 is expressed throughout early development. (H) Morpholino knock down of kermit2 in stage 11 embryos. 30 ng of control morpholino (CO 30) has no effect on Kermit2 expression. The Kermit morpholino at 10 ng and 20 ng (MO 10 and MO 20) decreases kermit2 expression below control levels. Molecular mass is indicated to the left of the panel. (A–C) size marker  = 200 µm. (D–F) size marker  = 30 µm.

**Figure 5:**
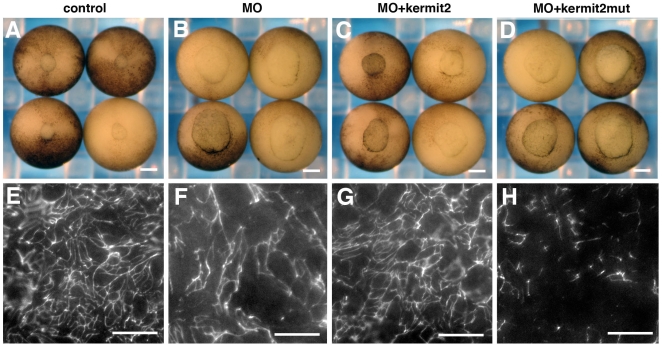
Expression of kermit2 mRNA rescues morpholino knock down of kermit2. (A–D) Ventral view of stage 12 embryos. (E–H) Late gastrula stage BCR stained for FN. (A) Stage 12 control embryos gastrulate normally and (E) assemble a dense FN matrix. Embryos injected with 10 ng of Kermit2 morpholino (MO; B) gastrulate normally, and (F) exhibit a small decrease in FN matrix assembly. (C) Embryos injected with kermit2 inhibiting morpholino and kermit2 mRNA (MO+kermit2) exhibit a small delay in blastopore closure and (G) a partial rescue of FN matrix assembly. (D) Embryos injected with the kermit2 morpholino and the dominant negative kermit2 construct (MO+kermit2mut) exhibit delayed blastopore closure and (H) significant reduction in FN matrix assembly. (A–D) size marker  = 200 µm. (E–H) size marker  = 30 µm.

We used morpholino knockdowns to further elucidate the role kermit2 plays in early development. A 5 mismatch control morpholino (CO) has minor effects on FN assembly that are not significant enough to block gastrulation (Figure 4B, E). The kermit2 morpholino (MO) inhibits FN assembly and blocks blastopore closure (Figure 4C, F). Western blots indicate GIPC is a maternal protein and expressed throughout all stages of early development (Figure 4G). The inhibiting morpholino reduces the amount of kermit2 protein, particularly in the post gastrula stages. In our hands injection of increasing amounts of morpholino resulted in no further decrease in kermit2 protein levels (Figure 4H) and increasing amounts of the control morpholino resulted in non-specific inhibition (not shown). Injection of kermit2 mRNA in the presence of the inhibiting morpholino rescues blastopore closure and FN matrix assembly (Figure 5C, G). Embryos co-injected with the morpholino and the dominant negative kermit2mut construct cannot close their blastopores and have very little FN on the BCR (Figure 5D, H). Similar to the results we obtained with the dominant negative construct, the defects in gastrulation are not due to disruptions of mesodermal genes or molecules involved in FN assembly (Supplemental material S 2). In our hands the dominant negative kermit2mut construct has a more consistent phenotype than the morpholino and we used this construct in most of our experiments.

**Figure 6:**
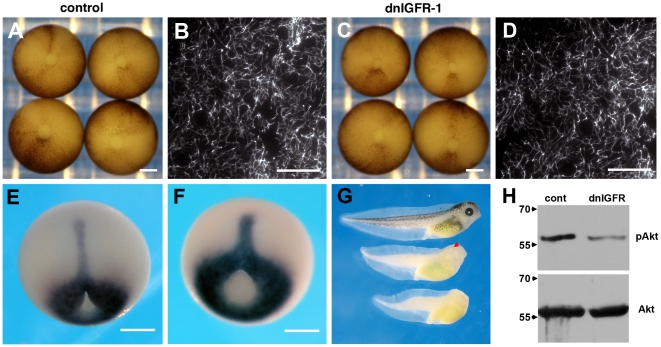
Inhibition of FN matrix assembly is not due to IGF signaling. (A, B) Control embryos close the blastopore by stage 12 and elaborate a dense FN matrix. (C, D) Embryos expressing a dominant negative IGFR-1 construct (dnIGFR-1) appear similar to control embryos and elaborate a dense FN matrix. (E) Xbra expression in control embryos. (F) Xbra patterning is not altered by blocking IGF signaling. There is a minor effect on axial extension that is clearly revealed in tadpoles (G) Control tadpoles (top) are longer than tadpoles resulting from embryos expressing dnIGFR-1 (middle). The dnIGFR-1 construct results in anterior defects including reduced or absent eyes (arrowhead). Tadpoles obtained from embryos that express kermit2_mut_ show severe anterior truncations and mesodermal defects (bottom). (H) Western blots demonstrating inhibition of IGF signaling by the dnIGFR-1 construct. The phosphorylation of Akt (pAkt) seen in controls (cont) is not maintained in animal caps that express the dominant negative IGFR-1 construct (dnIGFR). Bottom panel shows total Akt expression in the same lysate. Molecular mass is indicated to the left of the panel.

It has been shown that kermit2 binds the IGF receptor-1 and is required for IGF signaling in the oocyte [25] and neurula stage embryo [26]. Therefore the possibility existed that the lack of FN assembly resulted from a disruption of IGF signaling in the late blastula. When we express a dominant negative IGFR-1 construct [28]we observe no effect on FN matrix assembly or blastopore closure (Figure 6A–D). There is a reduction in notochord extension and we occasionally see a delay in blastopore closure at stage 11.5–12 (Figure 6F), however, by stage 13 embryos appear normal. The dnIGFR-1 construct produces eye and anterior neural defects that have been described previously [28] (arrowhead Figure 6G). This indicates that kermit2 plays essential roles in early embryogenesis and is required for FN matrix assembly outside of it's described role in regulating IGF signaling.

### Kermit2 Mediates Cell Migration

**Figure 7:**
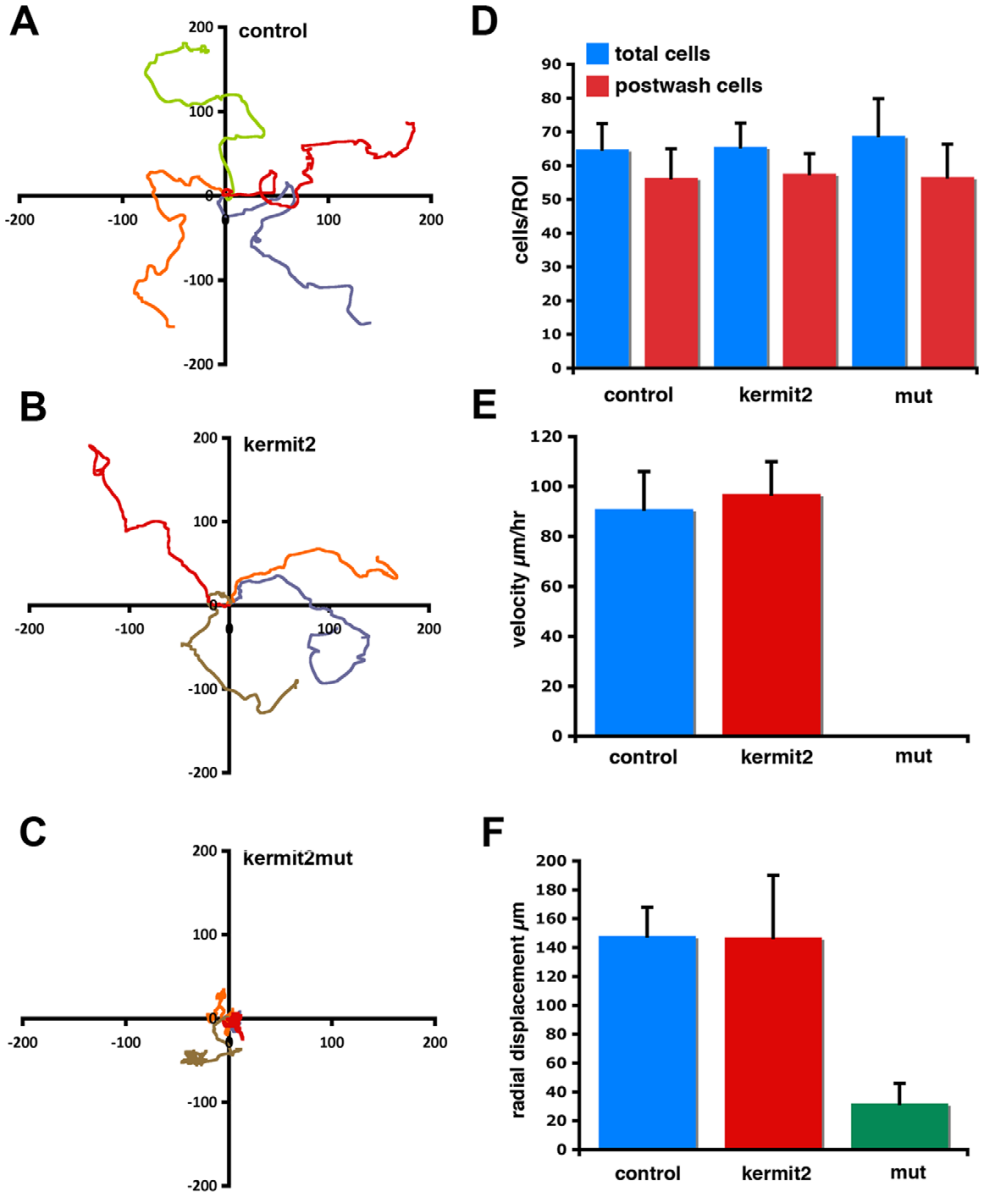
Cell adhesion assays. (A–C) Spider graphs representing migration tracks of individual cells plated on FN substrates. Each graph contains 4 representative tracks with the start point set to (0,0). Horizontal and vertical scale is in µM. (C) Kermit2_mut_ expressing cells have reduced migration paths as compared to (A) control and (B) kermit2 expressing cells. (D) Quantification of activin-treated cell adhesion to FN substrates. Cells were plated on FN substrates and counted pre-wash (blue) and after washing to remove non-adherent cells (red). Control, kermit2, and kermit2_mut_ expressing cells all show similar affinity for FN substrates. (E) Activin-treated cell migration rate mediated by kermit2. Average cell migration velocities were measured using the track cells function of Openlab. The value for each construct represents the average (±SD) of 45 cells from three spawnings. Cells expressing the kermit2_mut_ construct (0.6 µM/hr) migrate significantly (P<0.005) slower than control (91 µM/hr) or kermit2 (97 µM/hr) expressing cells. (F) The radial displacement of activin-treated cells on FN substrates. Measurements are from the same cells as represented in (E). Kermit2_mut_ expressing cells travel significantly (P<0.01) less distance (32 µM) than control (148 µM) or kermit2 expressing cells (147 µM).

Cells isolated from the animal cap region of *Xenopus* blastulae can attach to FN and upon exposure to activin spread and migrate [6], [29]. As the cytoplasmic domains of α integrin subunits mediate this behavior [14] we asked if kermit2 played a role in the activin promoted migration of animal cap cells on FN. Isolated animal cap cells were treated with 20 ng/ml activin, plated on FN substrates, and individual migration recorded. Cells derived from embryos injected with water migrate persistently and translocate significant distances (Figure 7A, E, F). Cells over-expressing kermit2 show similar migration patterns (Figure 7B, E, F), while cells expressing the kermit2mut construct show decreased migration distances remaining close to their original site of adhesion (Figure 7C, E, F). The decreased migration distances observed in kertmit2mut expressing cells is not due to a lack of adhesion as cells expressing either kermit2 construct adhere to FN equally well (Figure 7D). Control and kermit2 injected cells show similar average migratory velocities and radial displacements, while cells expressing the dominant negative kermit2mut show very little displacement and hence low velocities (Figure 7E, F). Thus, kermit2 is required for cell migration on, but not adhesion to, FN.

### Kermit2 and Integrin α5β1 Endocytosis

**Figure 8:**
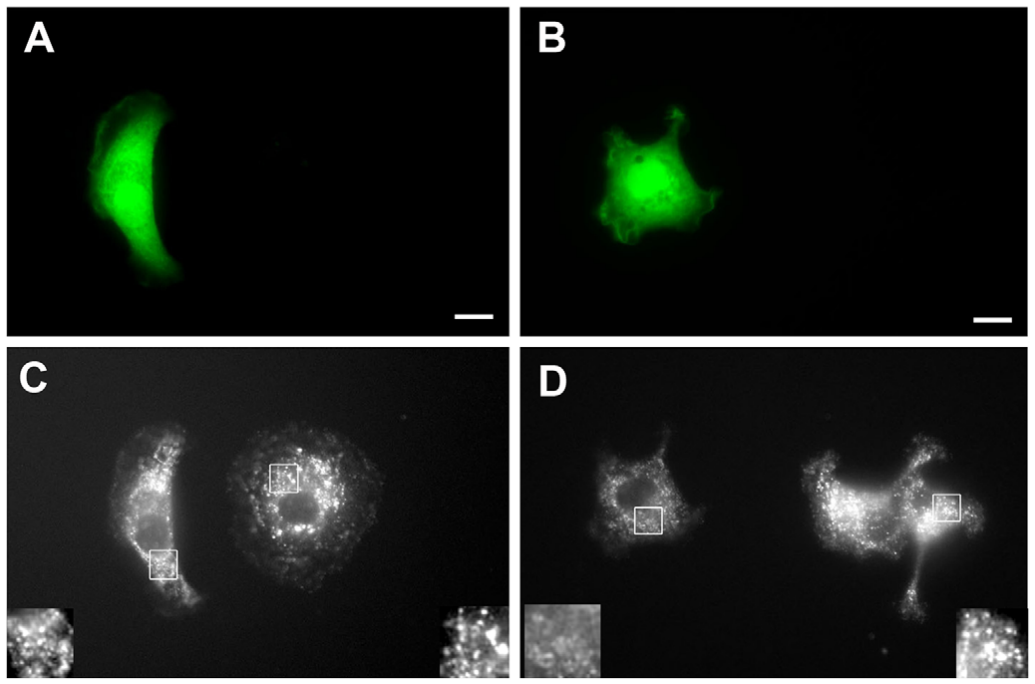
Kermit2 regulates internalization of antibody bound α5β1 integrin. *Xenopus* A6 cells were transfected with (A) GFP-tagged kermit2, or (B) GFP-tagged kermit2_mut_ and the endocytosis of antibody labeled α5β1 was estimated from fluorescent intensity using the density slice function of Openlab. (C, D) Staining of internalized α5β1 with fluorescent anti-mouse antibody. Non-transfected cells in the same dish act as controls. Insets in C and D represent 25 µM^2^ ROI's used to estimate pixel densities. (A, C) Kermit2 transfected cells have 827.3±23.0 pixels/ROI as compared to control cell ROI pixel densities of 841.5±13.3 pixels/ROI. (B, D) In kermit2_mut_ transfected cells the ROI pixel density is 530.8±51.2, while in non-transfected cells from the same dish have an average pixel density of 832.5±24.3 pixels/ROI. Pixel densities represent averages (±SD) from 10 individual cells from 4 separate transfections. Size marker  = 25 µM.

GIPC has a well-established role in the endocytosis of a number of transmembrane receptors. As integrin trafficking has been described as an essential component in cultured cell migration so we asked if kermit2 is involved in cell surface turnover of the α5β1 integrin during *Xenopus* embryonic cell migration. We initially approached this problem using a *Xenopus* tissue culture model. *Xenopus* kidney epithelial cells (A6 cells; ATCC # CCL 102) were transfected with GFP tagged kermit2 or kermit2mut. We obtain about 15–30% transfection rates and we have used un-transfected cells from the same plate as controls. We followed integrin α5β1 endocytosis by monitoring the internalization of the anti-α5β1 antibody P8D4 (Figure 8). In cells expressing the kermit2 construct significant quantities of α5β1 are found in cytoplasmic punctae (Figure 8A, C). In contrast cells expressing the kermit2mut construct show reduced levels of integrin endocytosis (Figure 8B, D). We quantified endocytosis using the measurements and density slice functions of Openlab on selected regions of interest (ROIs; Figure 8 insets). Images were collected at the same exposure and the same threshold values were used to quantify all images. Control and kermit2 expressing cells have similar rates of integrin endocytosis, while cells transfected with kermit2mut have fewer P8D4 positive vesicles than non-transfected cells in the same dish. This suggests that kermit2 plays a role in the endocytosis of integrin α5β1.

### α5β1 Endocytosis Lies Downstream Of Activin Signaling

**Figure 9:**
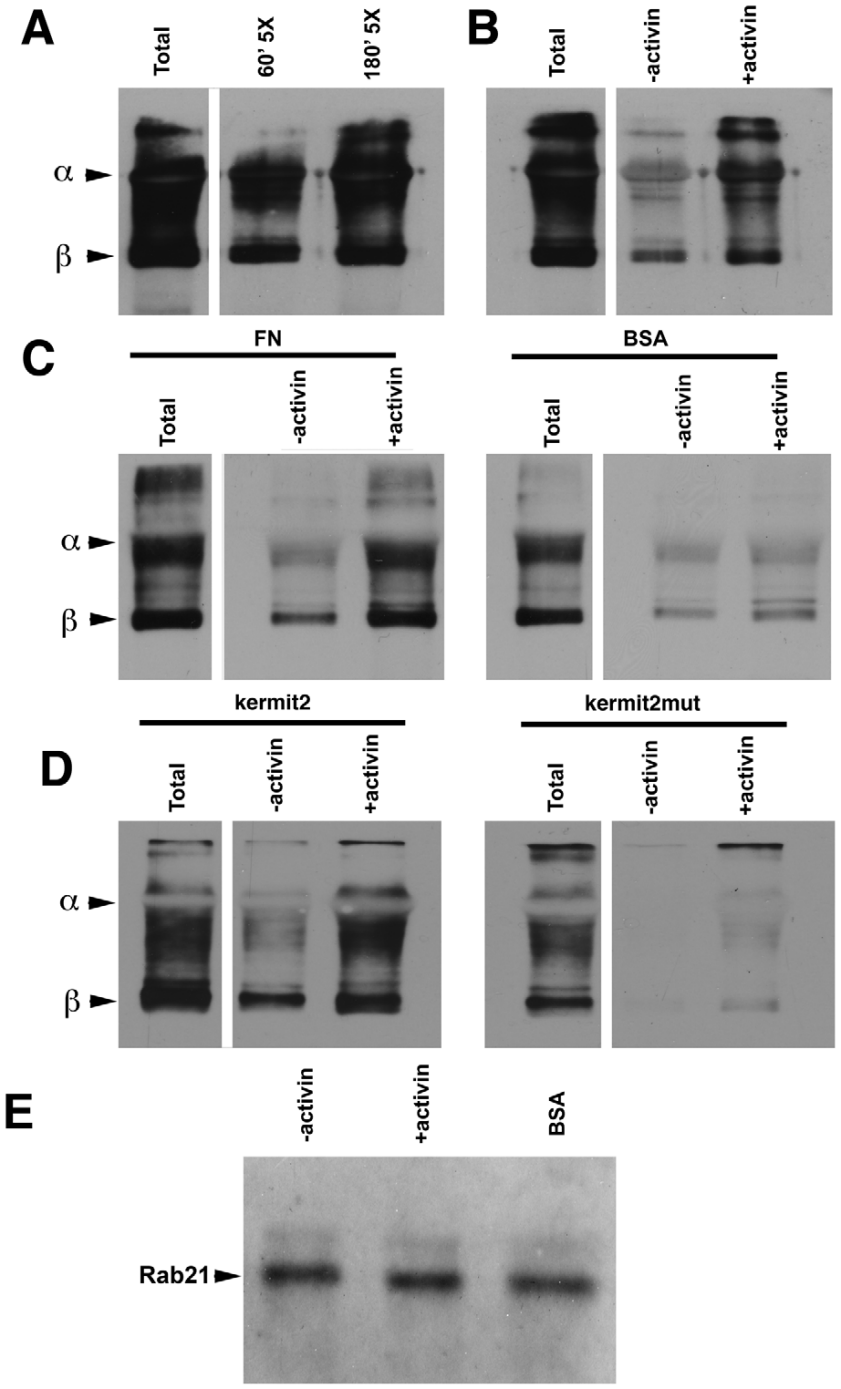
Endocytosis of α5β1 integrin. Cell surface α5β1 integrin was labeled with cleavable biotin and endocytosed integrin was immunoprecipitated and integrin subunits detected with streptavidin HRP on non-reducing western blots. α and β subunits are indicated to the left of the panels. The non-reduced P8D4 IgG used for the immunoprecipitation runs at the same molecular weight as the α subunit partially masking the signal. In all panels the total lane represents five fold more cells than represented in other lanes and the samples were not surface stripped. (A) Time course of α5β1 integrin endocytosis following activin induction. Increasing amounts of α5β1 are found in the cytoplasm at 60 minutes and 180 minutes following activin treatment. (B) α5β1 integrin endocytosis is stimulated by activin induction. Panels A&B come from the same gel and are separated for clarity. (C) Cell adhesion stimulates α5β1 endocytosis. Activin-tretaed cells adherent on FN substrates exhibit an increased rate of endocytosis as compared to cells on a non-adherent (BSA) substrate. (D) Kermit2 regulates α5β1 endocytosis. Activin-treated kermit2_mut_ expressing cells show reduced levels of α5β1 integrin endocytosis as compared to cells expressing kermit2. (E) Rab 21 coprecipitates with α5β1 integrin independent of mesoderm induction and adhesive substrate. Rab 21 was detected in α5β1 immunoprecipitates with A-14 antibody.

Having obtained evidence that kermit2 may be regulating α5β1 endocytosis we asked what role this may play in the embryo. Animal cap cells were surface labeled with cleavable biotin, induced with activin, and plated on FN. Once cells had spread and initiated migration surface biotin was stripped and intracellular α5β1 was immunoprecipitated with antibody P8D4. Increasing amounts of α5β1 are endocytosed over three hours, and cells that have been treated with activin show elevated levels of α5β1 endocytosis (Figure 9A, B). Adhesion to FN further influenced α5β1 endocytosis as cells adherent to FN show increased α5β1 endocytosis as compared to cells incubated on a non-adherent substrate (Figure 9C). Finally we asked if kermit2 played a role in activin mediated integrin endocytosis. Cells expressing kermit2 display similar patterns of α5β1 internalization as control cells (compare Figure 9C to 9B). Cells expressing the kermit2mut construct show decreased internalization of α5β1, in both animal cap cells and in animal cap cells exposed to activin (Figure 9D). These data suggest that integrin endocytosis is regulated by activin induction, cell substrate adhesion, and through interactions with kermit2.

As interactions with the Rab family of molecules has been implicated in integrin recycling we immunprecipitated α5β1 integrin and looked for coimmunoprecipitation of Rab 21. We found that Rab 21 appears to be constitutively associated with α5β1 independent of activin treatment and adhesion to FN (Figure 9). We found no association of integrin α5β1 with Rab5 (data not shown).

### Kermit2 And α5β1 Colocalize In Adherent Cells

**Figure 10:**
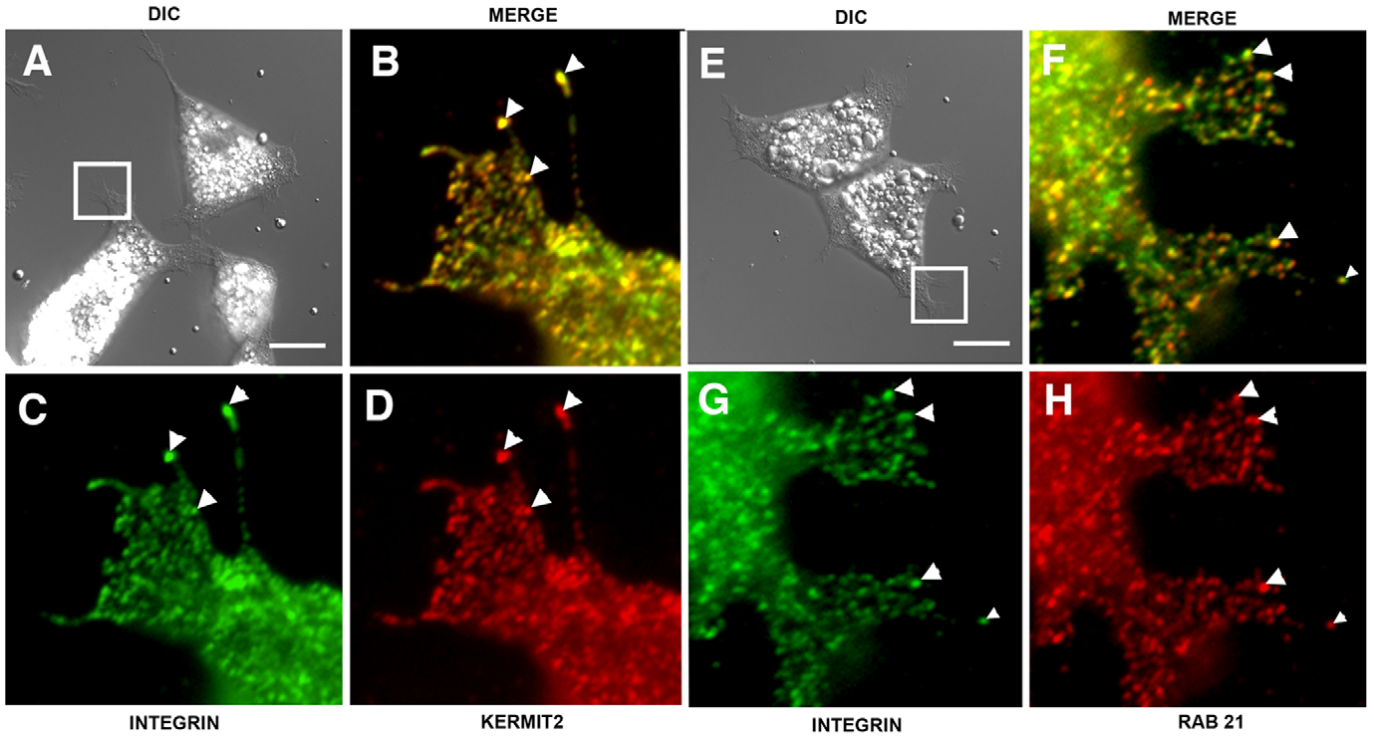
Colocalization of α5β1, kermit2, and Rab 21 in *Xenopus* embryonic cells. Activin-treated embryonic cells were plated on FN substrates and stained for α5β1 (green), or kermit2 (red D), or Rab 21 (red H). (A, E) DIC images of adherent cells, boxes represent areas magnified in B–D and F–H. (A–D) Integrin (C; green) and kermit2 (D; red) colocalize at sites of adhesion (arrowheads in B–D). (E–H) Rab 21 (H; red) and integrin (G; green) colocalize in embryonic cells (arrowheads in F–H). Staining of cells with secondary antibodies alone produced no detectable signal. Size marker  = 25 µM.

**Figure 11:**
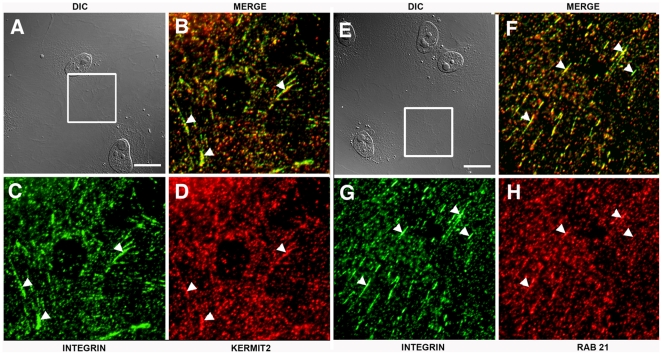
Colocalization of α5β1, kermit2, and Rab 21 in *Xenopus* XTC cells. XTC cells were stained for α5β1 (green), or kermit2 (red D), or Rab 21 (red H). (A, E) DIC images of adherent cells, boxes represent areas magnified in B–D and F–H. (A–D) Integrin (C; green) and kermit2 (D; red) colocalize in numerous vesicles and at focal adhesions (arrowheads in B–D). (E–H) Rab 21 (H; red) and integrin (G; green) colocalize in cytoplasmic vesicles and focal adhesions (arrowheads in F–H). Staining of cells with secondary antibodies alone produced no detectable signal. Size marker  = 25 µM.

Because kermit2 function is required for the temporal activation and endocytosis of integrin α5β1, we investigated the sub cellular localization of kermit2 and integrin α5β1 in animal cap cells migrating on FN. Animal cap cells were treated with activin and plated on FN substrates. Once the cells had spread and initiated migration they were fixed and stained with antibodies directed against α5β1, GIPC, and Rab 21. *Xenopus* embryonic cells are large and contain significant amounts of yolk (Figure 10A, E) making intra-cellular localization difficult. We decided to concentrate our analysis on lamellipodial and filopodial extensions as they are thin and do not contain yolk granules. In spread animal caps cells there is strong colocalization of integrin and kermit2 in filopodial extensions, and in small vesicles close the edge of the cell (arrows Figure 10B–D). Using the colocalization function in Openlab (Improvision) we obtain an average coefficient of colocalization (R) of 0.82+/−0.07 9 (N = 12) indicating a high correlation of fluorescent signal for integrin and kermit2. While there is a high correlation between integrin and kermit2 localization in filopodia and lamellipodia (67+/−14%) there are sites of integrin expression that are exclusive of kermit2. We then asked if Rab 21 played a role in α5β1 endocytosis in *Xenopus* cells. Colocalization of integrin α5β1 and Rab 21 indicate that there is significant overlap in the fluorescent signals (R = 0.49+/−0.03 (N = 12); Figure 10E–H). The association between integrin and Rab 21 takes place further back from the leading edge of the cell than that observed for kermit2 and integrin. As embryonic cells make poorly defined adhesive sites on FN substrates we asked if cultured *Xenopus* cells would exhibit similar interactions. In XTC cells [30] kermit2 and integrin α5β1 colocalize at focal adhesions as well as in vesicles proximal to sites of adhesion (Figure 11A–D). Similar to what we observe in embryonic cells, Rab 21 also colocalizes to these same sites (Figure 11E–H). Our results demonstrate that integrin α5β1, Rab 21, and kermit2 are localized to vesicles and sites of adhesion to FN in both embryonic and cultured cells.

## Discussion

Here we demonstrate that interactions between kermit2 and the cytoplasmic domain of integrin α5β1are essential to position-specific regulation of integrin function during *Xenopus* gastrulation. Furthermore, we report that kermit2 regulates the endocytosis of the α5β1 integrin downstream of activin signaling and that this internalization is required for cell motility on FN substrates. Our data support a model where inductive interactions promote α5β1endocytosis and this is a key step in the initiation of cell migration during gastrulation.

### Kermit2 Interacts With α5 and α6 Integrin Cytoplasmic Domains

GIPC has previously been shown to interact with the Type 1 PDZ recognition sequence in cytoplasmic domains of the α5, α6A and α6B integrin subunits [16]. Here we show a similar interaction exists in *Xenopus*. Kermit2 interacts strongly with both the α5 and α6 subunits that have a Type1 recognition sequence, but not with the αV subunit that lacks such a sequence. The α6 subunit contains a canonical Type I recognitions sequence, TSDA, and has the highest affinity for kermit2. The α5 subunit has a C-terminal ASEA sequence substituting the aspartic acid at position -2 with a conserved glutamic acid and also has a non-polar alanine in the place of polar threonine at position -3. Tani et al. (2001) have reported that substitution of a non-polar amino acid at position -3 in the human α6A cytoplasmic domain reduces the interactions with GIPC by 60%. This is similar to the difference in the affinity of the α5 and α6 domains that we observe in our two-hybrid assays.

While we found that there are substantial amounts of kermit2 in *Xenopus* embryos our GST pull downs and coimmunoprecipitations suggest that only a small fraction of kermit2 in the embryo is competent to bind to endogenous α5β1 integrin. Similarly, Liu et al. (2001) found that while GIPC is essential for steady surface expression of gp75, very little GIPC is found associated with gp75 in immunoprecipitates. A possible explanation for the low yield of GIPC in immunprecipitates was suggested by Versano et al. (2006) [18]. They demonstrate that the interaction between the nerve growth factor receptor, TrkA and GIPC is transient and restricted to the cell periphery. We also find that kermit2 associated with integrin α5β1 is predominantly found in vesicular structures that are proximal to sites of ECM adhesion in cultured cells, or localized to the periphery of embryonic cells. This restricted temporal and spatial sub-cellular window of interaction may well account for the low yields of GIPC in our IP's and pull down experiments. It is remarkable that while we see restricted interactions between kermit2 and α5β1, the dominant negative kermit2 construct is effective at abolishing cell migration. This may reflect the system we are using as *Xenopus* cells are large and display relatively small lamellipodial protrusions and hence have only a small adhesive footprint. Small differences in adhesion may well have dramatic overall effects on cell morphology. Interestingly, interfering with kermit2 function does not decrease cell adhesion but does eliminate lamellipodia and cell migration suggesting it may only be acting on activated α5β1 integrin. We do not have the reagents to directly assess α5β1activation, however activin-signaling drives increased endocytosis of α5β1in a kermit2 dependant manner, and the AEEL mutation in kermit2 interferes with embryonic cell behaviors that can be directly attributed to integrin activation.

### Kermit2 and Mesoderm Cell Migration

Our results demonstrate that in activin induced mesodermal cells kermit2 mediates changes in integrin α5β1 regulated cell motility. It is unclear as to how activin mediates changes in cell adhesion, however, previous evidence indicate these changes in cell motility may lie in a distinct pathway from activin induced mesodermal patterning [6], [31]. In the embryo activin participates with other TGF-β family members in the long-range patterning of the mesoderm, therefore it is unlikely that activin directly impinges on pathways that regulate cell adhesion. Recent evidence suggests that Xnr's and activin regulate distinct genes and that the primary role of activin may not be to pattern tissues but rather to regulate cell behaviors downstream of patterning [32]. In vitro activin activated changes in cell adhesion are coincident with timing of mesodermal marker expression and it is likely that activin regulates cell adhesion through the expression of molecules that influence integrin adhesion. The identity of these molecules remains unknown.

As kermit2 is a protein known to act in the endocytic pathway [33] it is unlikely that kermit2 is acting to directly activate integrins but rather regulating functional surface expression of activated integrins. Several studies have demonstrated that growth factor stimulation results in integrin endocytosis and recycling back to the membrane [34], [35]. Valembri et al. (2009) recently demonstrated that in endothelial cells VEGF stimulation drives interactions between GIPC and integrin α5β1that results in Rab 5 mediated endocytic recycling of the activated integrin. We predict a similar mechanism is operating in the region-specific activation of integrin in *Xenopus* as mesoderm induction in vitro results in kermit2-mediated endocytosis of Rab 21 associated α5β1.

Our observation that α5β1endocytosis is driven in part by adhesion to FN is in line with others that have described integrin endocytosis takes place at sites of adhesion [21], [36]–[39]. Embryonic *Xenopus* cells do not form clearly defined structures such as focal adhesions making description of adhesive sites difficult. However, at sites where we observe integrin accumulation we also see Rab 21/α5β1 and kermit2/α5β1positive vesicles. Moreover, in cultured cells we see accumulation of Rab 21/α5β1 and kermit2/α5β1 positive vesicles at focal adhesions. This suggests that there is a feed back loop between integrin activation, adhesion, and recycling functionally tying inside-out and outside-in integrin signaling. Valembri et al. (2009)implicate nrp1 in a complex with α5β1as a requirement for integrin endocytosis downstream of FN adhesion. We have not identified a specific partner protein in *Xenopus*, however, α5β1 immunoprecipitates from activin-induced adherent cells contain a 35 KDa protein that is biotin labeled at the cell surface and endocytosed with the α5β1integrin (Figure S3). Interestingly, we observe the opposite situation for αV containing integrins in which a 55 KDa protein coimmunoprecipitates in non-adherent cells and is lost in immunoprecipitates from adherent cells (supplemental material S3). We are presently pursuing the identity of these proteins and it is interesting to speculate that their presence is directly related to receptor activation and or recycling. Another possibility for the change in behavior we see following adhesion to FN is that integrin cross talk stimulates receptor endocytosis [40]. The αVβ3 integrin is expressed in the early embryo and perhaps it acts to regulate the recycling of the α5β1 receptor down stream of FN assembly in a manner similar to that described by White et al. (2007) [41]. Testing of these ideas awaits the development of a specific inhibitor for the αV containing integrins in *Xenopus*.

### FN Assembly vs. Cell Migration

Disrupting kermit2 interactions with α5β1affects both FN matrix assembly and mesendoderm cell migration without affecting the ability of cells to adhere to FN. While it is not difficult to reconcile integrin recycling with cell migration it is unclear what role kermit2 plays in FN matrix assembly. FN matrix assembly in *Xenopus* has been proposed to stem from increased tension across the blastocoel roof independent of integrin expression [42] suggesting stable surface expression of integrin α5β1 is required. Furthermore, the assembly of FN is inhibited by antibodies that block α5β1 interactions with the synergy site of FN indicating that activated integrin is essential for FN assembly [6]. FN can be assembled by chimeric integrins that do not recognize the synergy site, however, only the α6 and α5 cytoplasmic domains, that contain the Type I PDZ recognition motif recognized by kermit2, confer the ability to assemble FN matrix [14]. Since this critical cytoplasmic PDZ domain is essential it may be that kermit2 is required to export activated integrin α5β1 or retain integrin on the surface of pre-gastrula stage cells facilitating FN assembly. Such a temporally restricted role has been described in melanoma cells where GIPC is required to move gp75 from the golgi to the cell membrane [23]. Alternatively, the scaffolding role of kermit2 may be essential to the regulation of α5β1 mediated FN assembly. Experiments that decrease expression of GIPC in endothelial cells prevent FN matrix assembly, a process dependant on GIPC mediated interactions between α5β1 and nrp1 [21].

### Multiple Roles For kermit2

Previous publications using morpholino knockdowns did not describe an early role for kermit2 in *Xenopus* development. However, it is clear in these studies that MO knockdowns produce a variety of phenotypes in morphant embryos, including deficits in axial extension [26]. Our experiments with morpholinos also demonstrate there is considerable variation in the axial extension of morphant embryos, although the defects in anterior neural and eye development are always robust. We show that kermit2 is present as maternal protein and morpholino knockdowns only reduce kermit2 protein levels by about 50% through gastrulation. When we combine the MO with the dominant negative construct embryos resemble those obtained with the dominant negative construct alone suggesting the MO only partially inhibits kermit2 function during gastrulation. This indicates that kermit2 protein levels are partially regulated through translation of zygotic transcripts but that maternal protein is likely still functional during gastrulation. Our interpretation of these observations is that kermit2 regulates cell surface turnover over of a variety of receptors, including α5β1 integrin. The morpholino begins to impinge on kermit2 expression at gastrulation when α5β1 regulates both FN assembly as well as cell movements [13]. As there is considerable variation in the timing of FN assembly and mesoderm movements in individual *Xenopus* embryos, the morpholinos may only affect a subset of embryos in which these processes occur relatively late and maternal kermit2 expression cannot compensate for the morpholino inhibition of zygotic kermit2 expression. This would produce embryos in which FN assembly and cell movements are inhibited resulting in a truncated AP axis. In embryos in which FN assembly and cell movements take place earlier, the morpholino would have less effect and maternal kermit2 is present in high enough levels to traffic integrin. These embryos would show a more dominant anterior neural defect and less axial defects. While speculative such a scenario is supported by the observation that the dominant negative construct affects not only FN assembly and cell movements, but also anterior neural development later in embryogenesis. Due to the promiscuous binding of kermit2 to many known target molecules we cannot completely eliminate the possibility that the defects we observe are due to multiple interactions. Despite this it is clear that kermit2 can regulate integrin activity, and that the dominant negative construct produce embryo phenotypes that closely resemble those obtained when FN assembly [13], [43] and integrin function are inhibited [44].

There is a closely related Kermit molecule expressed in *Xenopus* embryos that regulates neural crest induction [45]. The similarity between kermit and kermit2 brings up the possibility that the kermit2mut construct inhibits the function of kermit. The design of our dominant negative construct blocks interactions between the PDZ binding domain in kermit2 and the PDZ domain of its partners and therefore should have no inhibitory effect on kermit function. Furthermore, kermit/GIPC family members act through the linking of protein complexes outside of the PDZ binding domain and the diversity in sequence between Kermit and kermit2 indicates there should not be any functional redundancy. Indeed, there is no evidence for redundant roles for closely related kermit proteins [26].

In *Xenopus* kermit2 was originally isolated as a binding partner for the IGF receptor [25] and has been implicated in regulating IGF signaling through interactions with the IGF receptor [26]. As IGF is known as a potent regulator of integrin function the possibility existed that our observations stemmed from disruptions in IGF signaling [46], [47]. A dominant negative IGFR previously shown to block IGF signaling had no effect on FN assembly. We see minor delays in blastopore closure and axial extension, however, by the end of gastrulation embryos appear normal. This suggests that our results with kermit2 do not stem from an inhibition of IGF signaling. Previously kermit2 has been described as acting primarily in the anterior neural plate. It is interesting that the strongest interaction we described in yeast two-hybrid assays is between kermit2 and the α6 integrin subunit that is also expressed in the developing central nervous system. We are now looking at the potential interactions between the α6β1 integrin, IGFR-1, and kermit2 in the neural plate.

In mammals GIPC is known to regulate TGF-β signaling through interactions with the type III receptor [48]. A type III receptor homologue has not been characterized in *Xenopus* and we see no effect on mesoderm patterning or gene expression levels indicating that it is unlikely that our results stem from disruptions in TGF-β family member signaling.

In summary we propose that kermit2 is involved in the region specific activation of integrin α5β1. Here we demonstrate that kermit2 interacts with the α5β1 integrin and that this interaction is required for receptor endocytosis and cell migration. Kermit2 function is mediated partially through activin signaling, although previous evidence suggests this in unlikely to be directly linked to mesoderm patterning. As receptor endocytosis is also influenced by interactions with FN our results suggest that kermit2 is at the nexus of several signaling pathways regulating integrin recycling. The turnover of α5β1 may be required for keeping a population of activated integrins on the surface of cells during gastrulation, a time when several integrin-mediated events are of importance. These include both inside-out signaling during FN assembly, as well as outside-in signaling during cellular rearrangements.

## Materials and Methods

### Ethics Statement

All animals were handled in strict accordance with good animal practice as defined by the University of Waterloo Office of Research Ethics. All animal work was approved by the University of Waterloo Animal Care Committee under AUPP #09-12.

### Embryo Culture

*Xenopus laevis* adults were purchased from Nasco (Fort Atkinson, Wisconsin). Embryos were obtained by standard methods [49]. Embryos were staged according to Nieuwkoop and Faber (1967) [50]. Prior to injection fertilized embryos were dejellied in 2% cysteine. Embryos and explants were imaged using a Zeiss Lumar Stereoscope using axiovision software (Zeiss).

### DNA Constructs and Morpholinos

A full-length cDNA representing *Xenopus* XGIPC/kermit2 (Accession AAL58320) in pCS2+ [51] was obtained as a gift from X. Liu (Ottawa Health Research Institute). Site-directed mutagenesis was used to alter the amino acid sequence ALGL, within the PDZ domain of kermit2, to AAEL, generating the kermit2_mut_ construct. Kermit2 and kermit2_mut_ constructs were subcloned into the EcoRI and BamHI sites of pEGFP-N1 (gift from J. Miller; University of Minnesota). mRNA was produced *in vitro* by cutting CS2+ plasmids with *Not* 1 and transcribing with SP6 polymerase using standard methods. mRNA was purified on Mega Clear columns (Ambion).

Kermit2 and kermit2_mut_ were subcloned into pJG4-6 [52] using EcoR1 and Xho1 restriction sites. Cytoplasmic domains of the *Xenopus* α5, α6 and αV subunits were isolated by PCR using primers described previously [14]. The cytoplasmic domains of the α5, α6, and αV subunits were subcloned into pEG202 [52] using EcoR1 and Xho1 restrictions sites. All subclones were confirmed by sequencing.

GST constructs were as described in [53]. Morpholinos were obtained from Gene Tools LLC. Kermit 2 morpholino sequence: AGAGGCATCTTTCTTTCAGCGAAGG. Kermit2 5 place mis-match morpholino: AGAcGCATgTTTgTTTCAGCcAAcG.

Gene expression levels were estimated using RT-PCR. mRNA was isolated from embryos using standard methods [54]. RT-PCR was performed on single stranded cDNA using the following primers: EF1α forward CAGATTGGT GCTGGATATGC, reverse ACTGCCTTGATGACTCCTAG. Chordin forward AACTGCCAGGACTGGATGGT, reverse GGCAGGATTTAGAGTTGCTTC. Xbra forward GGA TCG TTA TCA CCT CTG, reverse GTG TAG TCT GTA GCA GCA. FN forward CCCTCAATGGTGTAGCCAAAAC, reverse TGAACTCCTTCTCTGGACCGTG. Integrin α5 forward TGTTCTACATCCACATCCCTTGC, reverse AAAGTCATTTCCACTGAGCAGACC. Integrin β1 forward TGGTTGGAGAAATGTCACTCGC, reverse AACACTTCCTTCCGTC TTCCCC. PCR products were quantified using ImageJ.

### Immunofluorescence

Embryos were cultured until stage 12 and fixed in 2% trichloroacetic acid. Fixed embryos were washed in TBS/0.1% Tween 20 (TBST) and animal caps were excised. Animal caps were stained with monoclonal antibody 4B12 directed against FN [5] in TBST containing 1 µg/ml of BSA. Primary antibodies were detected using Alexa Fluor 488 Conjugated Goat Anti-mouse secondary antibody (Invitrogen). Animal caps were mounted on glass slides and imaged using a Zeiss Axiovert 200 microscope equipped with a Ludl motorized stage and Qimaging Retiga 1494 digital camera using Openlab imaging software (Perkin Elmer). FN assembly was qualitatively estimated from the density of fibrils found lining the blastocoel roof.

Animal caps were excised from stage 8 embryos and dissociated in Ca^2+^ Mg^2+^ free MBS. Cells isolated from embryos expressing kermit2 or kermit2_mut_ were either treated with 20 ng/ml Activin-A (R&D systems) or mock treated with water and plated on FN substrates in modified Stern's saline (MSS). Once sibling embryos had reached stage 10.5 attached cells were fixed for15 minutes in 4% paraformaldehyde, washed and stained with antibodies against integrin α5β1 (P8D4, gift from DW DeSimone), Rab 21 (Santa Cruz Biotech), or GIPC (C-20; Santa Cruz Biotech), Alexa 488 and 594 secondary antibodies were from Invitrogen. The C-20 anti-GIPC antibody was affinity purified against *Xenopus* kermit2. Cells were imaged and images were processed using the deconvolution and colocalization function of Openlab (Improvision). The value R is independent of signal intensity and background. Therefore R is not a quantitative estimate of molecular colocalization but an estimate of the dependency of spatial colocalization.

### Cell Migration Assays

Isolated animal cap cells were obtained from embryos expressing kermit2-GFP or kermit2_mut_-GFP and treated with activin as described previously [42]. Dissociated cells were plated on 50 ug/ml FN (Calbiochem) coated dishes in the presence or absence of 20 units/ml activin [55]. Cells expressing microinjected constructs were identified using GFP-expression. The cell migrations were monitored using a Zeiss Axiovert 200 microscope as described above and migrations tracks monitored using Openlab software (Perkin Elmer). At the end of the assay cells were fixed 30 minutes using 4% paraformaldehyde in MSS and imaged using DIC illumination. For cell attachment assays cells were isolated and plated as described above. Fields of view were recorded using a 5X objective on a Zeiss Axiovert 200. The plates were washed and fields of view recorded postwash. Cells in each field of view were counted.

### Animal Cap Extension Assays

Animal caps of embryos microinjected with kermit2 and kermit2_mut_ mRNA were excised from stage 8 embryos as described above. Animal caps from experimental and control embryos were cultured in 0.5X MBS in the presence or absence of 20 units/ml activin. As a control for normal development, sibling embryos were cultured in 0.1X MBS solution. Overnight explant extension were imaged using a Zeiss Lumar stereoscope.

### In Situ Localization

In situ localization was performed using standard methods [56] using a probe against Xbra [57].

### Yeast Two-Hybrid Assays

GIPC and α integrin subunit fusion constructs were used to cotransform DY1 *Saccharomyces cerevisiae* cells [58], which contains the β-Galactosidase reporter plasmid, pSH18–34. A positive control of known prey-bait interaction DY-1 ((pSH18–34)(pJG4-6-Rad53)(pEG202-Dbf4(FL)) was a gift from Dr. Bernard Duncker (University of Waterloo) and was used to standardize β-galactosidase activity. Transformants were grown in synthetic complete (SC) media plates lacking uracil, tryptophan, and histidine at 30°C to a concentration of 5×10^6^ cells/ml. Cells were washed and resuspended in 2% galactose-1% raffinose and lacking uracil, tryptophan, and histidine for 6 hours to induce prey expression [58]. Following induction, 5×10^6^ cells were harvested and the interactions between fusion proteins were quantified using ONPG [59]. Two colonies of each transformant pair were assessed and assays were preformed in triplicate. The expression of bait and prey fusions was confirmed by western blotting as described previously [60]. Prey fusion constructs were detected with antibody 12CA5 (Roche), and prey fusion constructs detected with a rabbit polyclonal serum directed against Lex-A (Sigma). Blots were visualized using HRP conjugated secondary antibodies (Jackson Labs), the ECL system (GE Healthcare; Mississauga, ON) and exposure to RXB x-ray film (Labscientific).

### Immunoprecipitations and Western Blotting

Embryo lysates were prepared from embryos homogenized in ESB (20 mM Tris (pH 7.5), 140 mM NaCl, 10 mM glycerol, 2 mM sodium-orthovanadate, 25 mM NaF, 1% Nonidet P-40), and 1X Complete Protease Inhibitor (Roche). For immunoprecipitates (IPs), antibodies (8C8 [61]), (P8D4 [44]), (P3C12 [62]) were conjugated to Protein G PLUS/Protein A-Agar beads (Protein G/A; Calbiochem). Embryo lysate was diluted 1∶3 in ESB and pre-cleared with Protein G/A beads. Embryo lysates were incubated for 3 hrs with antibody bound protein G/A beads at 4°C. Protein G/A complexes were washed four times in cold ESB. Immunoprecipitates were subjected to Western blotting using standard procedures. HA- tagged XGIPC was detected using antibody 12CA5 (Roche) and HRP conjugated anti-mouse secondary antibodies (Jackson Labs).

### Cell Culture

*Xenopus* A6 cells (ATCC# CCL-102) and XTC cells were maintained in 66% L-15 media (Sigma) supplemented with 10% FBS, 1% L-glutamine, 1% Penicillin/Streptomycin, 1% sodium pyruvate (Wisent). Cells were grown to 60–80% confluence before being transfected with 1.0 µg of purified plasmid using LipoFectamine (Invitrogen). Transfected cells were replated on 60 mm glass bottom dishes and imaged as described above.

### Receptor Endocytosis

Integrin internalization was monitored in A6 cells that were transfected with kermit2 and kermit2_mut_. Transfected cells were incubated at 4°C to slow integrin turnover. Cells at 4°C were incubated with anti-α5β1 antibody (P8D4) for 1 hour. Cells were washed three times with 66% L-15 medium to remove unbound antibodies and then incubated at room temperature to allow for normal cell membrane dynamics. Cells were fixed as described previously and blocked in TBS, 0.1% Triton X-100 and 1% Lamb Serum. Primary antibody was detected using a goat anti-mouse secondary antibody conjugated to Alexa Fluor 594 (Invitrogen) for 1 hour. Transfected cells were identified by GFP expression and imaged as described above. Images were processed using the density slice function to threshold images and vesicles counted using the measurements function of Openlab (Perkin Elmer). Object counts from regions of interest (ROIs) were used as estimates of integrin endocytosis.

Integrin endocytosis was evaluated in embryonic cells using cleavable biotin. Animal cap cells were obtained and dissociated as described above. Cell surface proteins were labeled on ice with 0.5 mg/ml sulfo NHS-SS-biotin (Thermo) in MSS- for 30 minutes. Cells were rinsed with ice cold 10 mM Glycine and subsequently treated with 20 units/ml activin in MSS- for 20 minutes at 14°C. Cells were rinsed repeatedly and plated on FN substrates as described above. After control embryos had reached stage 10.5 (approximately 3 hours) cell surface biotin was cleaved with 50 mM reduced Glutathione, 75 mm NaOH in MSS for 30 minutes on ice. Cells were rinsed with ice cold MSS and remaining free sulfhydryl groups were alkylated with 5 mg/ml iodoacetamide in MSS. Cells were rinsed repeatedly in MSS, and lysed in ESB. Protein extracts were immunoprecipitated with MAb P8D4 and P3C12 as described above. Retrieved proteins were separated by non-reducing SDS-PAGE and blotted to nitrocellulose. Biotin-labeled proteins were detected with HRP conjugated streptavidin (GE Healthcare) as described above.

## Supporting Information

Figure S1 Western blots of bait and prey fusion protein constructs used in yeast two hybrid assays. Figure shows prey and bait combinations from replicates (top and bottom row) from a single assay. Western reveals that expression there is approximately equal expression of bait and prey.
(0.09 MB TIF)

Figure S2 Inhibition of kermit2 function does not affect gene expression. (A,B) RNA was isolated from stage 10.5 embryos that were injected with water, kermit2 mRNA or kermit2mut mRNA (mut). (A) Mesoderm induction is unaffected by kermit2 construct expression. mRNA levels were quantified using RT-PCR with primers specific to EF1Î± (blue), chordin (red) and Xbra (yellow). (B) Expression levels of FN (blue), integrin Î±5 (red), and integrin Î^2^1 (yellow) subunits are unaffected by kermit2 construct expression. Expression of the kermit2 or kermit2mut construct has no effect of transcript abundance. (C) Morpholino knock down of kermit2 does not affect mesoderm patterning. (C,D) RT-PCR on stage 10.5 embryos that had been injected with water, control morpholino (COMO), or inhibiting morpholino (MO). (C) There is no effect on mRNA abundance for EF1Î± (blue), chordin (red) and Xbra (yellow). (D) Morpholino knockdown of Kermit2 does not alter the expression of FN (blue), integrin Î±5 (red), and integrin Î^2^1 (yellow) subunits. Expression levels were standardized to control embryos (control) in all experiments (N = 3) error bars represent standard deviations.
(0.50 MB TIF)

Figure S3 Immunoprecipitation of biotin labeled cell surface integrins. In the Î±5Î^2^1 immunprecipitation a cell surface protein of 30 kD co-precipitates strongly in samples adherent to FN (arrowhead). The Î±5Î^2^1 immunoprecipitation is the same as Figure 7. In the Î±VÎ^2^3 immunprecipitations the same 30 kD band is visible but is not specific to FN adherent cells (arrowhead). A 50kD band appears in non-adherent cells.
(0.45 MB TIF)
