# Correction: Integrin α5β1 Function Is Regulated by XGIPC/kermit2 Mediated Endocytosis during *Xenopus laevis* Gastrulation

**DOI:** 10.1371/journal.pone.0143904

**Published:** 2015-11-25

**Authors:** Erin Spicer, Catherine Suckert, Hyder Al-Attar, Mungo Marsden

Panels B and D of Fig 6 are mistakenly from the same sample. The authors have provided a corrected version of Fig 6 here, which includes new images for both panels. These revised panels are from distinct samples than those used in the original figure. The uncropped images used to create the revised panels can be viewed as Supporting Information. The authors confirm that this error does not alter their results.

**Fig 6:**
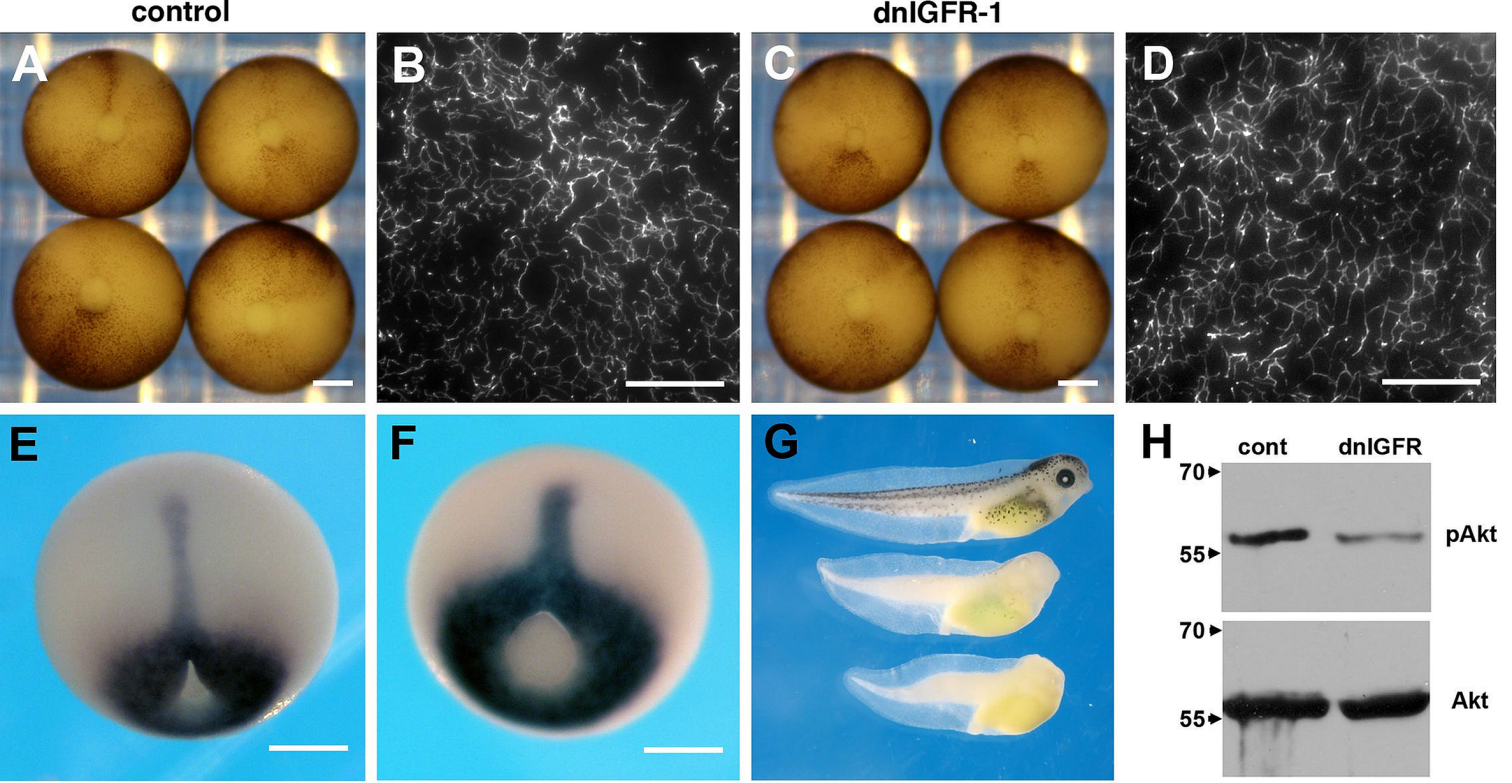
Inhibition of FN matrix assembly is not due to IGF signaling. (A, B) Control embryos close the blastopore by stage 12 and elaborate a dense FN matrix. (C, D) Embryos expressing a dominant negative IGFR-1 construct (dnIGFR-1) appear similar to control embryos and elaborate a dense FN matrix. (E) Xbra expression in control embryos. (F) Xbra patterning is not altered by blocking IGF signaling. There is a minor effect on axial extension that is clearly revealed in tadpoles (G) Control tadpoles (top) are longer than tadpoles resulting from embryos expressing dnIGFR-1 (middle). The dnIGFR-1 construct results in anterior defects including reduced or absent eyes (arrowhead). Tadpoles obtained from embryos that express kermit2_mut_ show severe anterior truncations and mesodermal defects (bottom). (H) Western blots demonstrating inhibition of IGF signaling by the dnIGFR-1 construct. The phosphorylation of Akt (pAkt) seen in controls (cont) is not maintained in animal caps that express the dominant negative IGFR-1 construct (dnIGFR). Bottom panel shows total Akt expression in the same lysate. Molecular mass is indicated to the left of the panel.

## Supporting Information

S1 Fig Original.tif file used to create the revised Panel B. (TIF)

S2 Fig Original.tif file used to create the revised Panel D. (TIF)
