# Supplementary figures and images for: Integrin α5β1 Function Is Regulated by XGIPC/kermit2 Mediated Endocytosis during Xenopus laevis Gastrulation
Source: PLoS One. 2010 May 17;5(5):e10665. doi: 10.1371/journal.pone.0010665 (PMC2871791; doi:10.1371/journal.pone.0010665)

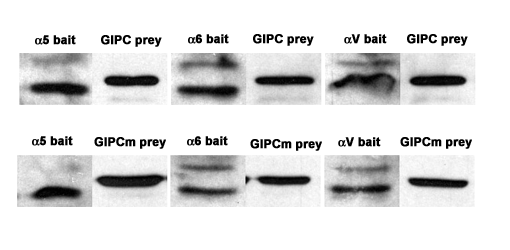

Supplement: Figure S1 — Western blots of bait and prey fusion protein constructs used in yeast two hybrid assays. Figure shows prey and bait combinations from replicates (top and bottom row) from a single assay. Western reveals that expression there is approximately equal expression of bait and prey. (0.09 MB TIF) [file pone.0010665.s001.tif]

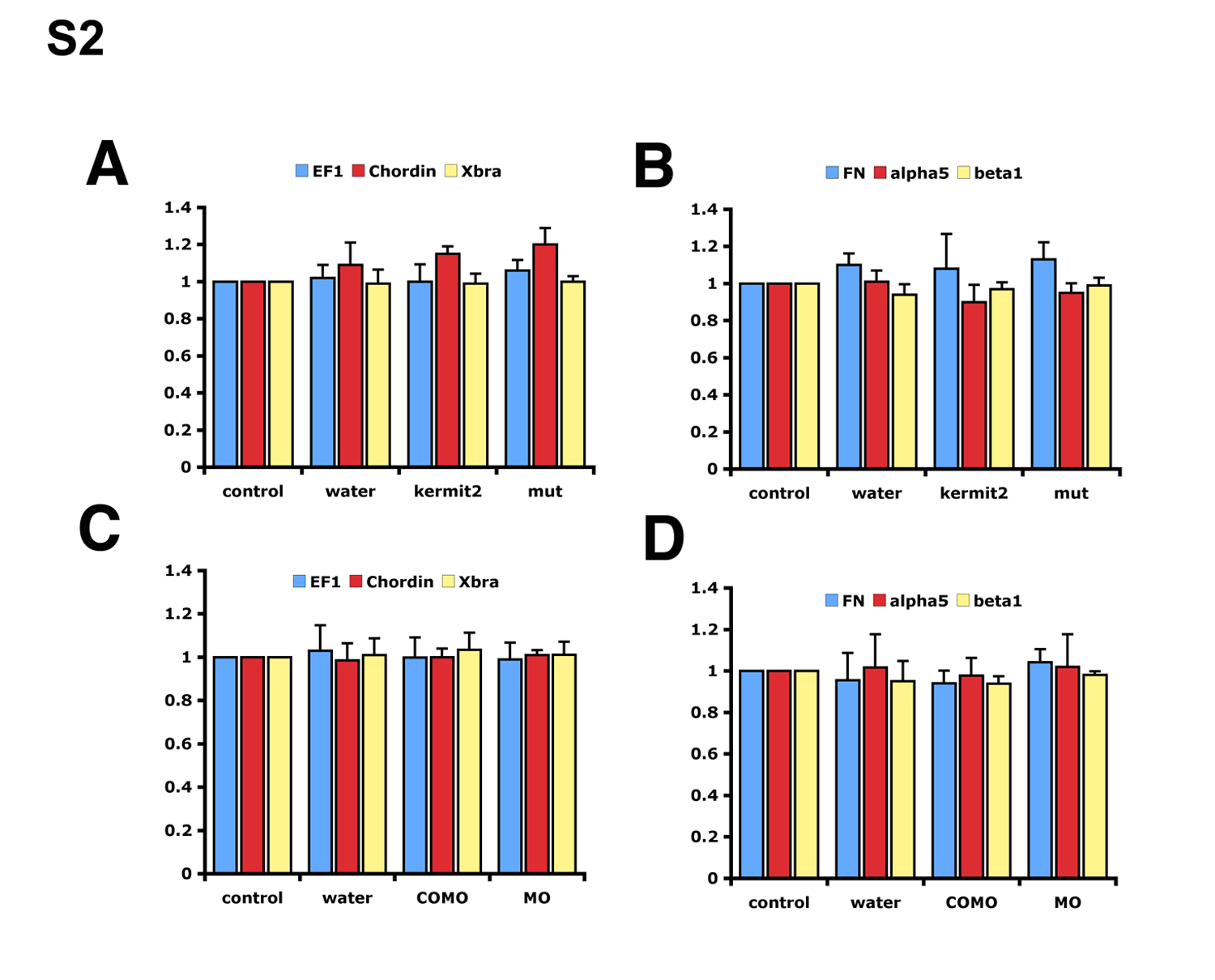

Supplement: Figure S2 — Inhibition of kermit2 function does not affect gene expression. (A,B) RNA was isolated from stage 10.5 embryos that were injected with water, kermit2 mRNA or kermit2mut mRNA (mut). (A) Mesoderm induction is unaffected by kermit2 construct expression. mRNA levels were quantified using RT-PCR with primers specific to EF1Î± (blue), chordin (red) and Xbra (yellow). (B) Expression levels of FN (blue), integrin Î±5 (red), and integrin Î21 (yellow) subunits are unaffected by kermit2 construct expression. Expression of the kermit2 or kermit2mut construct has no effect of transcript abundance. (C) Morpholino knock down of kermit2 does not affect mesoderm patterning. (C,D) RT-PCR on stage 10.5 embryos that had been injected with water, control morpholino (COMO), or inhibiting morpholino (MO). (C) There is no effect on mRNA abundance for EF1Î± (blue), chordin (red) and Xbra (yellow). (D) Morpholino knockdown of Kermit2 does not alter the expression of FN (blue), integrin Î±5 (red), and integrin Î21 (yellow) subunits. Expression levels were standardized to control embryos (control) in all experiments (N = 3) error bars represent standard deviations. (0.50 MB TIF) [file pone.0010665.s002.tif]

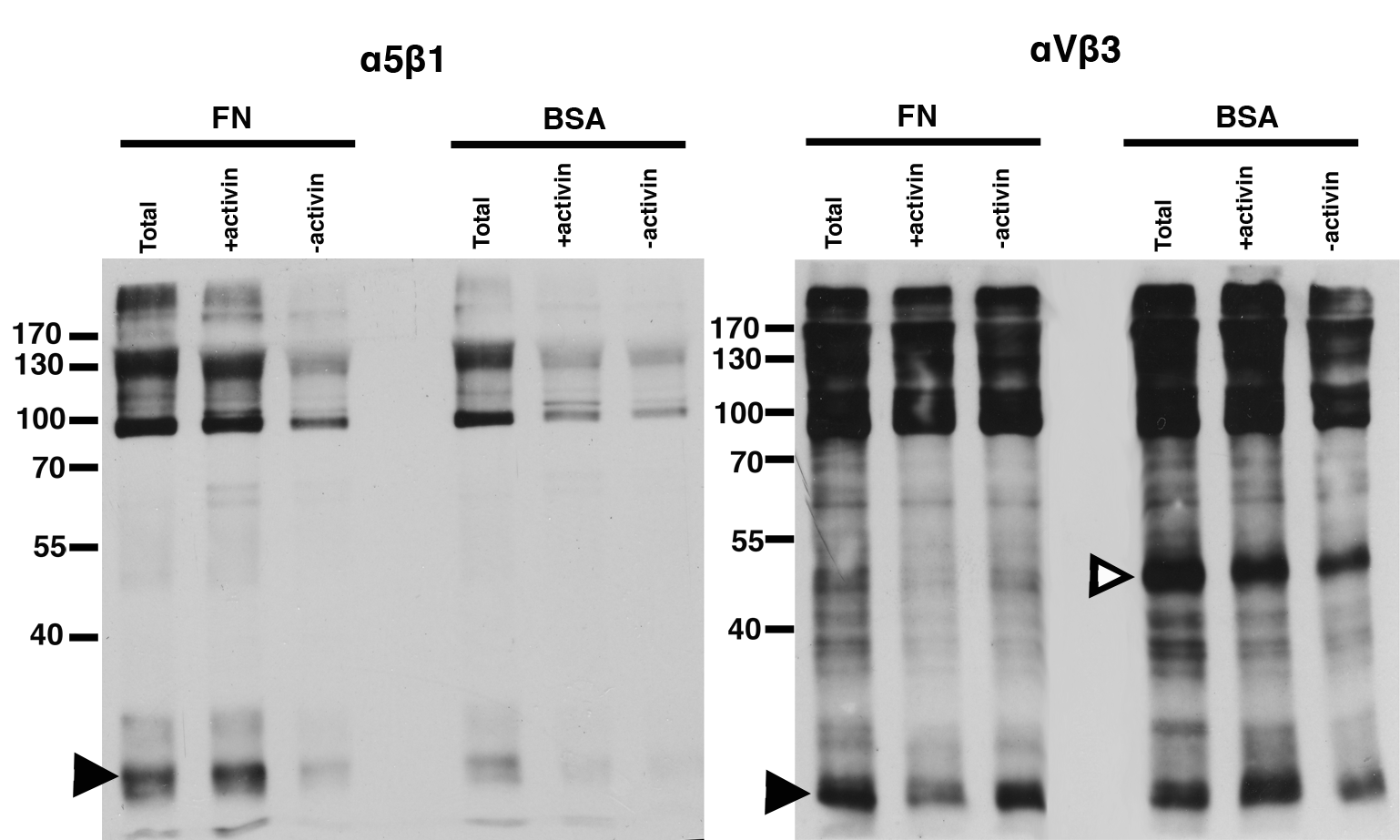

Supplement: Figure S3 — Immunoprecipitation of biotin labeled cell surface integrins. In the Î±5Î21 immunprecipitation a cell surface protein of 30 kD co-precipitates strongly in samples adherent to FN (arrowhead). The Î±5Î21 immunoprecipitation is the same as Figure 7. In the Î±VÎ23 immunprecipitations the same 30 kD band is visible but is not specific to FN adherent cells (arrowhead). A 50kD band appears in non-adherent cells. (0.45 MB TIF) [file pone.0010665.s003.tif]
