# Supplementary figures and images for: Correction: Integrin α5β1 Function Is Regulated by XGIPC/kermit2 Mediated Endocytosis during Xenopus laevis Gastrulation
Source: PLoS One. 2015 Nov 25;10(11):e0143904. doi: 10.1371/journal.pone.0143904 (PMC4659680; doi:10.1371/journal.pone.0143904)

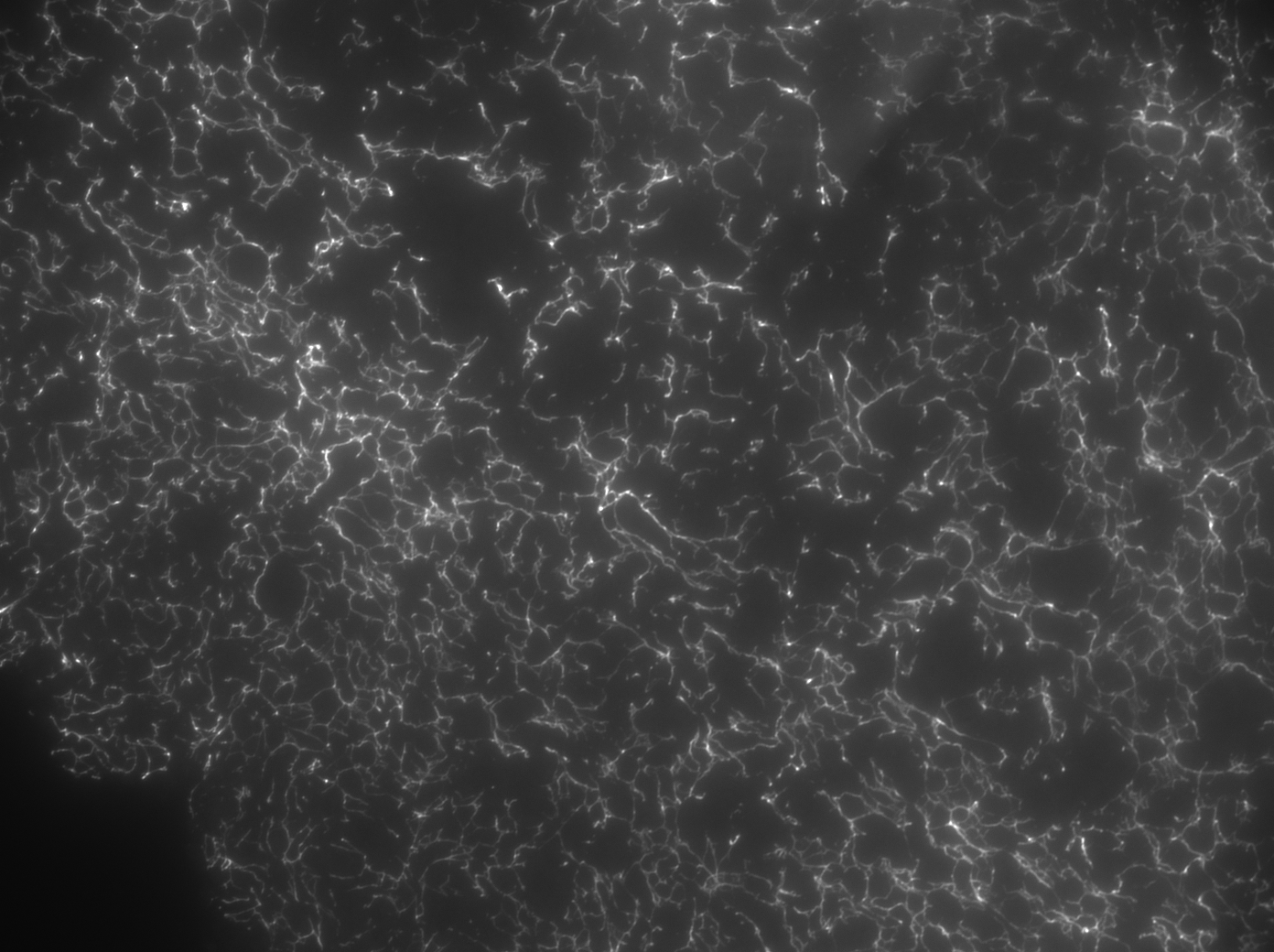

Supplement: S1 Fig — (TIF) [file pone.0143904.s001.tif]

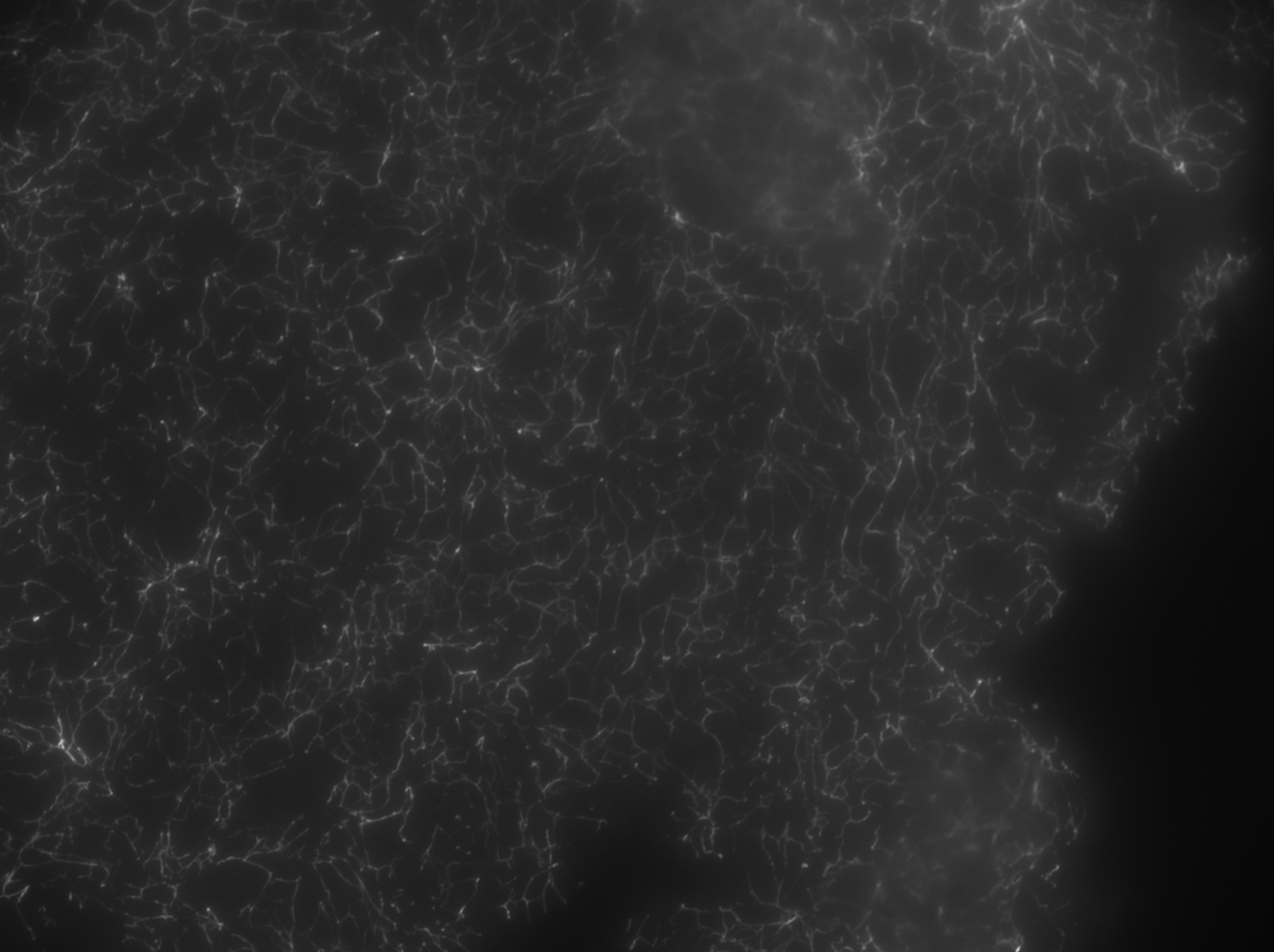

Supplement: S2 Fig — (TIF) [file pone.0143904.s002.tif]
